# Facile Synthesis of Cauliflower Leaves Biochar at Low Temperature in the Air Atmosphere for Cu(II) and Pb(II) Removal from Water

**DOI:** 10.3390/ma13143163

**Published:** 2020-07-15

**Authors:** Qilong Ge, Qi Tian, Muhammad Moeen, Sufang Wang

**Affiliations:** 1 College of Environmental Science and Engineering, Taiyuan University of Technology, Taiyuan 030024, China; geqilong0108@link.tyut.edu.cn (Q.G.); moeenejaz79@gmail.com (M.M.); wangsufang@tyut.edu.cn (S.W.); 2 Department of Environmental Science and Engineering, Taiyuan College, Taiyuan 030032, China; 3 College of Civil Engineering, Taiyuan University of Technology, Taiyuan 030024, China

**Keywords:** biochar, vegetable waste, low temperature, preparation mechanism, heavy metal removal, sorption mechanism

## Abstract

In this study, a facile and low-cost method for biochar (CLB) preparation from vegetable waste (cauliflower leaves) was developed at a low temperature (120 °C) in the air atmosphere. The prepared mechanism, adsorption mechanism, and performance of CLB for Cu(II) and Pb(II) sorption were investigated using Scanning electron microscopy- energy dispersive X-ray spectroscopy(SEM-EDS), X-ray diffraction(XRD), Fourier transform infrared spectroscopy(FTIR), and a series of sorption experiments. Then the CLB was subjected to single and double element sorption studies to examine the effect of pH value on the Cu(II)/Pb(II) sorption capacities and then competitive sorption priority. There are both more hydroxyl (–OH) and carboxyl (–COOH) functional groups on the surface of CLB compared to those from control (without H_3_PO_4_ impregnation), resulting in more ion exchanges and complexation reaction for CLB with Cu(II) and Pb(II). Besides, the phosphorus-containing groups (e.g., P = OOH, P = O.), which newly formed with H_3_PO_4_ impregnation, could also enhance sorption, especially for Pb(II), this way leaded to its adsorption and precipitation as Pb_5_(PO_4_)_3_OH crystals. The performance of maximum adsorption capacities of CLB toward Cu(II) and Pb(II) were 81.43 and 224.60 mg/g, respectively. This sorption was slightly pH-dependent, except that the sorption capacity improved significantly as the pH value of the solution increased from 2 to 4. Competitive sorption experiment confirmed that Pb(II) had a higher sorption priority than Cu(II).

## 1. Introduction

During the past decades, heavy metals contamination of surface and underground water poses a significant threat to humans and aquatic organisms because they cause high carcinogenicity and toxicity [1]. These toxic and non-biodegradable metals tend to exist persistently in the ecosystem and accumulate quickly in the organism, such as human bodies, when they exceed the permissible limit [2,3]. Cu(II) and Pb(II) are among these most toxic metals. The significant effect of copper ions results in gastrointestinal depression, lungs cancer, liver and kidney damages. Excess amount of lead causes central nervous system damage, infertility, and anemia [4]. So developing new approaches to remove heavy metals, especially Cu(II) and Pb(II), from wastewater efficiently and selectively is a prior and significant step for the effective control of heavy metals pollution.

Among many different technologies of heavy metals removal from wastewater (e.g., ion exchange, precipitation, membrane filtration, and sorption), sorption procedure is the most welcome because it is cost-effective and eco-friendly [5]. Compared to other adsorbents, the feedstock of biochar is abundant and renewable, biomass produced from food and agricultural wastes could be used as biochar precursor, which will reduce the discarded quantity and prevent environmental pollution [6,7]. So, biochar is considered as one of the most promising absorbents. Considering the limited sorption capacity of original biochar, much attention has been given to find new approaches to modify the surface physicochemical property of pristine biochar for enhancing its adsorption capacity [8,9,10].

However, surrounding biochar prepared by the conventional methods, such as high-temperature pyrolysis (350–750 °C), hydrothermal carbonization (200–350 °C), several drawbacks arise. One of the main disadvantages of these methods is that they need high temperatures, generally more than 200 °C, which means considerable environmental burden and much electricity consumption [11]. Besides, compared with biochar prepared under high temperature, the yields of biochar made under relatively low temperature can be improved, as thermal decomposition of some constituents of biomass, unusually small molecular organic matter, will take place under high temperature [12]. Another defect is to provide the anaerobic (or oxygen-limited) condition; most biochar preparation methods require a closed environment. Pyrolysis, for example, often involves filling a tube furnace with nitrogen [13,14]. The hydrothermal method always needs to use a closed reactor and autoclave [15,16]. Although the use of acid (particularly H_3_PO_4_) can increase the performance of biochar and some experiments about H_3_PO_4_ modified biochars have been carried out in inert atmosphere at higher temperatures [17,18,19,20,21], very few literatures has reported about biochar preparation under the condition of less than 200 °C, especially under air atmosphere, because biochar prepared under inert atmosphere means a complex and costly preparation process. Furthermore, H_3_PO_4_ is environmentally friendly due to its non-toxicity and easily being washed by water [19]. Thereby the biochar preparation method at low temperature under the air atmosphere will become more convenient and more productive. Moreover, the mechanisms of biochar preparation and its metal ions adsorption, influence factors of metal ions sorption capacity need to be further examined.

In addition, vegetable wastes, an important type of residues, occur in large quantities in food and beverage activities, wholesale markets and agricultural activities. Because of transport and disposal costs, the production of vegetable wastes increases the costs of market operation [22]. Because they are rich in cellulose and lignin, they are the preferred feedstock for biochar production. Due to this background, cauliflower leaves, one of the typical and highly produced vegetable wastes from cultivation and food industries, was carbonized with H_3_PO_4_ at low temperature in the air to prepare cauliflower leaves biochar (CLB). The main aims of this work are (1) to prepare the CLB at 120 °C under air condition; (2) to explore the prepared mechanism of H_3_PO_4_ for the physiochemical properties of CLB surface during carbonization process; (3) to investigate the removal mechanism and capacities of Cu(II) and Pb(II) by CLB in aqueous solution; (4) to evaluate the influence of pH value on the sorption capacity of CLB and competitive relationship between Cu(II) and Pb(II).

## 2. Materials and Methods

### 2.1. Experimental Chemical Reagents and Materials

Cauliflower leaves were collected from farmer’s market of Taiyuan (37°48′6″ N, 112°35′46″ E, Shanxi, China). All analytical grade chemical reagents (e.g., Cu(NO_3_)_2_·3H_2_O, H_3_PO_4_ (85 wt.%), Pb(NO_3_)_2_, NaNO_3_, NaOH, and HCl) were purchased from Hengxing Chemical Preparation Corporation, Tianjin, China. Deionized water (DW, 18.7 MΩ) was used to prepare chemical solutions.

### 2.2. Biochar Preparation

**Figure 1:**
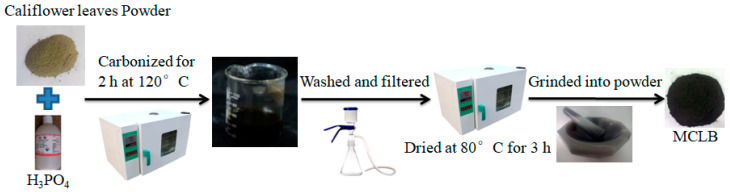
Preparation process of cauliflower leaves biochar (CLB) from cauliflower leaves.

To remove dust and soluble impurities, cauliflower leaves were firstly washed several times with DW. The leaves were kept in an oven for drying at 80 °C until their color turned pale yellow (about 24 h). After that, they were crushed and sieved with 20-mesh screens. Subsequently, 5 g of the dry cauliflower leaves was added in 50 mL of 42.5% H_3_PO_4_solution and then stirred. The mixture was carbonized in the air-dry oven for 2 h at 120 °C under air atmosphere. Finally, the treated leaves were washed with DW to get rid of excess H_3_PO_4_, filtered and dried at 80 °C for 3 h, then ground into powder and labeled as CLB for further utilization. The biochar prepared with similar procedures without H_3_PO_4_ addition marked as CL. The detailed process is shown in Figure 1.

### 2.3. Biochar Characterizations

Element compositions of pristine cauliflower leaves (PCL), CL and CLB were measured with an Elemental Analyzer (Vario EL cube, Elementar, Hamburg, Germany). CHNS and O modes of operation were selected respectively to determine the corresponding element content. Using the modified dry-ashing method [23], the content of major inorganic elements was identified by an inductively coupled plasma optical emission spectroscopy (ICP-OES) (Agilent 730, Palo Alto, CA, USA). After mixing the biochar sample with DW (1:10 *w:v*) and shaking them for 1 h in a mechanical shaker, the pH value was measured using a pH meter (Lei-ci PHS-3C, Shanghai, China). To measure specific surface areas (SSA), the surface area analyzer (ASAP2020C, Norcross, GA, USA) was used by the tests of N_2_ adsorption-desorption isotherm experiments using BET (Brunauer–Emmet–Teller) method. Based on the method introduced by Johnson et al. (1996) [24], the zeta potential of biochar samples was measured by zeta potentiometric analyzer (HOBRIBA Sz-100, Kyoto, Japan) after they were sonicated in DW for 10 min. The surface morphology of CL, CLB, and CLB loaded with Cu(II)/Pb(II) was investigated at 15 kV voltage by using scanning electron microscopy (SEM, JSM-IT200, Tokyo, Japan) equipped with an energy dispersive X-ray spectroscopy (EDS). EDS was used to identify the composition of the elements of biochars’ surfaces. After shaking the mixture of biochar and 0.3 mM Cu(II)/Pb(II) (0.25:100 *w*:*v*) for 24 h at 25 ± 1 °C, post-sorption samples were filtered by membrane filters (pore size = 0.22 μm), washed several times with DW and dried at 80 °C in an oven for overnight.XRD patterns of pre- and post-samples were checked by X-ray diffractometer (XRD, ULTIMA IV, Tokyo, Japan) with Cu Kα radiation (λ = 1.5418 Å), 2θ range from 5° to 60° at the rate of 8°/min, 40 mA electrical current, 40 kV voltage. After pressing the mixture of sample and spectroscopic grade KBr (1:100 *w:w*) into a flake, the change of surface functional group of the samples before and after metal adsorptionwas determined by Fourier transform infrared spectrometer (FTIR, TENSOR27, Karlsruhe, Germany). The FTIR spectra of sample were recorded in the spectrum ranged from 400 to 4000 cm^−1^. The surface chemical compositions of sample were measured by an X-ray photoelectron spectrometer (XPS, ESCALAB 250Xi, New York, NY, USA).

### 2.4. Adsorption Kinetics and Isotherm Experiments

Generally, samples were prepared with 0.25 g CLB with 100 mL solution containing 200 and 600 mg/L of Cu(II) and Pb(II), respectively, in Erlenmeyer flasks, adsorption kinetics experiment was carried out without pH adjustment. These mixtures were taken back at various time intervals with a wide range between 0.05 and 48 h when the biochar suspensions were shaken at 25 ± 1 °C. The adsorption isotherm experiments were performed under the same temperature using different concentrations of Cu(II) (30–300 mg/L) and Pb(II) (50–800 mg/L), respectively. Adsorbent and adsorbate (0.25:100 *w*:*v*) were also withdrawn after shaking for 24 h. NaNO_3_ solution (0.01 M) was selected as a background electrolyte performed. The blank experiment was carried out without CLB to distinguish between possible heavy metals precipitation and actual sorption mechanisms. After all the mixtures were filtrated, Cu(II) and Pb(II) concentrations in the filtrate were determined by ICP. The metal ions adsorption capacity was calculated using the following equation:

(1) $$q_{e}=\frac{(C_{0}-C_{e})V}{m}$$

In which, *q*_e_, *C*_0_, *C*_e_, *V,* and *m* represent the metal ions sorption amount perunit mass of sorbent (mg/g), initial metal ions concentration (mg/L), equilibrium metal ions concentration (mg/L), the solution volume (L), and the mass of sorbent (g), respectively.

### 2.5. Influence of Initial pH

The effects of initial pH on the interactions between adsorbent and adsorbate were studied by adjusting the initial pH value of 200 mg/L Cu(II)/Pb(II) solution to 2–7 with 0.1 MHCl and NaOH [17]. An amount of 0.5 g CLB and 200 mL adsorbate solutions were mixed in Erlenmeyer flasks, shaken for 24 h, filtered, and measured for Cu(II)/Pb(II) concentration in the filtrate.

### 2.6. Competitive Adsorption

The competitive adsorption experiments were done by dual-metal solution of Cu(II) and Pb(II). An amount of 0.5 g CLBwas added into200 mL sorbate solutions containing 0, 40, 80, 120, 160, 200 mg/L (0, 0.625, 1.25, 1.875, 2.5, 3.125 mM) Cu(II) with 200 mg/L (0.966 mM) Pb(II) as background solution, contrariwise including 0, 40, 80, 120, 160, 200 mg/L (0, 0.193, 0.386, 0.579, 0.773, 0.966 mM) Pb(II) with 200 mg/L (3.125 mM) Cu(II) as background solution. The treatment of equilibrium mixtures was the same as the adsorption experiment described above.

### 2.7. Adsorption and Desorption Experiments

To evaluate the reusability of CLB, adsorption and desorption experiments were conducted. An amount of 0.25 g CLB was used to treat 100 mL solution including 200 mg/L Cu(II) and 600 mg/L Pb(II), respectively, at pH 6. It was then shaken for 24 h in a mechanical shaker at 25 ± 1 °C. 10 mL of the solution was collected by centrifugation at 8000 rpm for 15 min. The filtrate was collected. After measuring the metals concentration, desorption experiments were carried out with 0.1 mol/LHNO_3_ for 60 min. After filtering and drying at 105 °C for 12 h, the recovered CLB was used to adsorb metals ions under the same condition described as the previous adsorption experiments.

### 2.8. Statistical Analyses

In this study, Origin software (8.0, Electronic Arts Inc., Redwood, CA, USA) was used to perform allfigures. All experiments were performed in duplicate and additional studies were carried out when two measurements varied at more than 5%.

## 3. Results and Discussion

### 3.1. Characterization and Preparation Mechanism of CLB

Table 1 presented the elemental composition, specific surface area, pH and pH_zpc_ values of PCL, CL, and CLB. It can be observed that carbon (C), hydrogen (H), and oxygen (O) were the primary elements for all of them. Because fresh pristine cauliflower leaves contained a high content of moisture, there was a large quantity of H and O elements. Lots of H_2_O existing in the leaves cellcould dilute the phosphoric acid concentration. Then functionality of the protons was inhibited [17]. So, drying pristine cauliflower leaves to remove moisture was of great necessity. Other common biochar materials contain similar elemental contents as the CLB, which indicated that a biochar sorbent was successfully prepared at 120 °C carbonization temperature under the air atmosphere [12,25]. CLB contained higher elemental carbon than CL, while the oxygen and hydrogen content of CLB was lower than that of CL, leading to the lower ratio of H/C and O/C of CLB. Because dehydration reaction that can remove oxygen and hydrogen is one of the primary mechanisms, the degree of this reaction can be promoted by the phosphoric acid [25]. Besides, a large quantity of oxygen and hydrogen in the CL sample indicated that alarge amount of oxygen-containing groups existed inside or beside the carbon skeleton, which could also be proved in the FTIR spectra discussed later. In addition, the slight growth of the carbon content of CLB was possible as a result of the effects of phosphoric acid on organic carbonization and preservation of small molecular species in the biochar [17]. Moreover, phosphorus element content (1.77%) of CLB went up probably because phosphorous groups were strongly adsorbed on the surface of biochar and some chemical bonds (e.g., C–O–P, P–O–P), during the carbonization process, were likely to be newly formed.

The specific surface area (SSA) of CLB was 55.42 m^2^/g, which was much higher than that of CL. Zeta potential greatly affected the electrostatic interaction between the biochar and heavy metals ions. pH_zpc_ value of CLB and CL were presented in Table 1, indicating that the surfaces of CLB were charged negatively because the pH value (4.56) of CLB was more than its pH_zpc_ (3.27).

H_3_PO_4_ could seep into the cracks of lignin of cauliflower leaves and mix well with them. During high-temperature activation, water vapor would be formed via catalytic dehydration reaction between H_3_PO_4_ and lignin. In addition, H_3_PO_4_ could easily react with oxygen-containing functional groups which were thermally unstable and could decompose to CO_2_ and H_2_O (g). The reaction equation was:

10C + 4H_3_PO_4_→10CO + P_4_ + 6H_2_O (2)

So, lots of pores were created [26]. Besides, as the oxygen-containing phosphoric acid occupied the internal volume of the material, the developed pore structure could be obtained after washing the material. Moreover, catalytic dehydration of H_3_PO_4_ resulted in that oxygen-containing groups fixed on the surface and internal pore walls of CLB. The pore size decreased and even transferred from mesopores to micropores. Therefore, the SSA of CLB increased (Table 1) [19].

**Figure 2:**
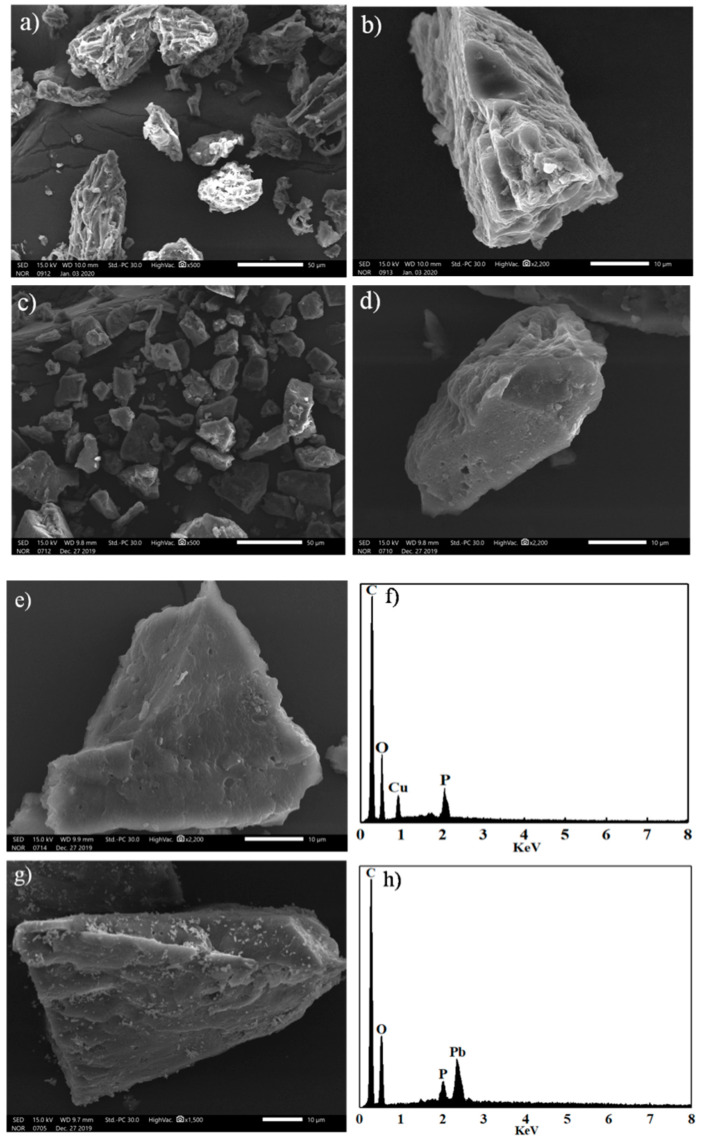
Scanning electron microscopy (SEM) images of CL (**a**,**b**), CLB (**c**,**d**) with different magnification, and CLB after 0.3 mM Cu(II) (**e**) and 0.3 mM Pb(II) (**g**) adsorption, respectively, with corresponding energy dispersive X-ray spectroscopy (EDS) (Cu(II) (**f**), Pb(II) (**h**)).

**Figure 3:**
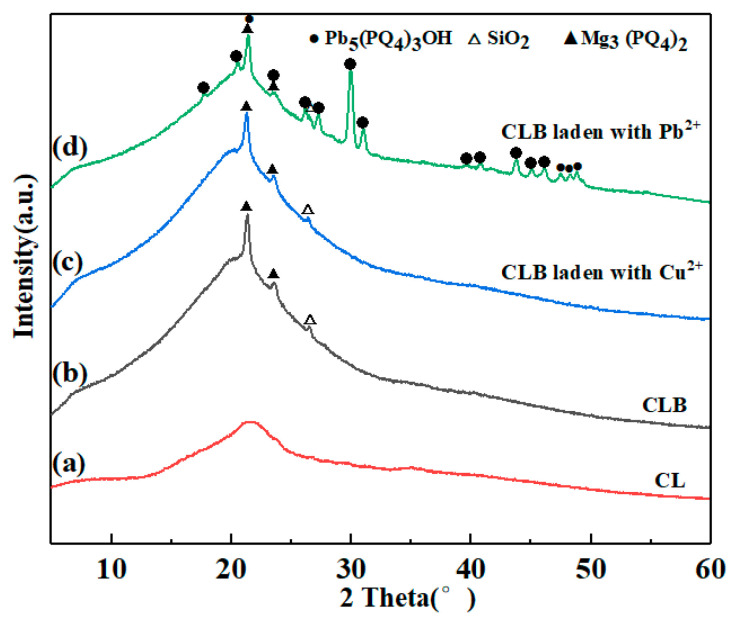
X-ray diffraction(XRD) patterns of CL (**a**), CLB (**b**) and CLB impregnated with 0.3 mM Cu(II) (**c**) and 0.3 mM Pb(II) (**d**) in single system.

The morphologies of biochars were examined by SEM. As seen in the image (Figure 2a,b), the conglobate and rough structure was recognized in CL. After carbonization with H_3_PO_4_, conglobate and rough structure disappeared. Instead, a block structure with a smooth surface was formed (Figure 2c,d). Besides, compared with the XRD pattern of CL (Figure 3a), a broader and stronger band centered at around 22° was detected (Figure 3b), confirming that the content of amorphous carbon after H_3_PO_4_ impregnation was larger than that without impregnation. Microspore formation was probably via the insertion of phosphorous functional groups into the carbon lattice, resulting in the amplification of amorphous form and lattice defect of the carbon structure. Meanwhile, crystalline minerals (e.g., magnesium phosphate (JCPDS card no. 43-0025) and silicon dioxide (JCPDS card no. 85-0798)) were formed.

**Figure 4:**
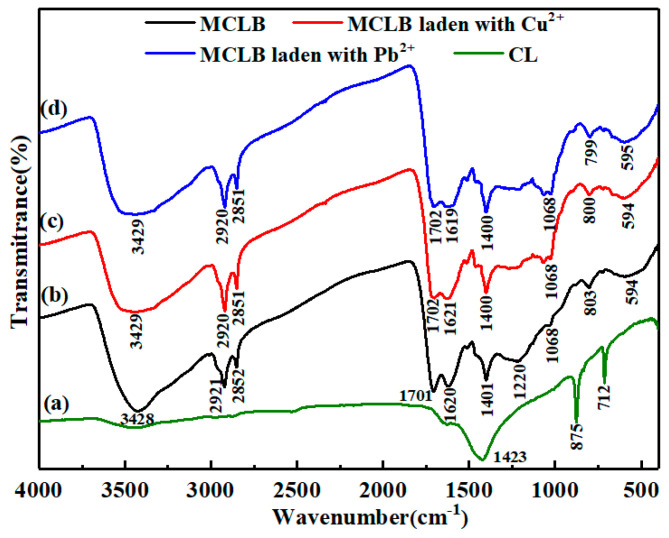
Fourier transform infrared spectra(FTIR) of CL (**a**) and CLB (**b**) before and after 0.3 mM Cu(II) (**c**) and 0.3 mM Pb(II) (**d**) adsorption, respectively.

Additionally, the FTIR spectra (Figure 4a,b) presented that original functional groups covering torsion around the C=C bond, carbonate, ethanol lignin (at about 712, 875, 1423 cm^−1^, respectively) disappeared and new functional groups including P = OOH and P = O occurred (at approximately 1220 cm^−1^) during the preparation process, compared with the functional groups of CLB [27,28]. Elemental and XRD analysis suggested that P and O contents of CLB were both higher than that of CL (Table 1 and Figure 3). Some previous literature also reported that carbonyl-containing groups and phosphorus-containing groups occurred or increased using the H_3_PO_4_ to activate carbon under anaerobic conditions at relatively high temperatures [19,29]. Besides, during the activation process, the stable structures, including aromatic C=C bonds (at 1620 cm^−1^), formed because of unsaturated bonds breaking. The aromatic ring could serve as donors, and the C=C bonds were significant for Cu(II)/Pb(II) sorption since these bonds can react with Cu(II) and Pb(II) via metal-π interaction [30,31]. The electron density of CL may weaker than that of C=C bonds of CLB, so Cu(II)/Pb(II)-π could be enhanced, which theoretically resulted in higher sorption of Cu(II)/Pb(II) onto CLB.

### 3.2. Cu(II) and Pb(II) Removal Mechanisms of CLB

Generally, the physical adsorption, which depends on porous structure and surface energy, has great effects on heavy metal ions’ storing performance of biochar. Although the SSA value of CLB was much higher than that of CL, CLB still had a much lower SSA value, compared with other popular adsorbent, such as activated carbon and zeolite. Physical adsorption might not be a primary mechanism of heavy metal adsorption. Hence, the chemical adsorption might dominate the adsorption properties of CLB. The pH values of CLB and CL were also detected. As other literature reported, biochars averagely prepared from pyrolysis usually showed weak alkaline [32]. However, CLB prepared via H_3_PO_4_ impregnation at 120 °C under air condition turned out to be weak acidic. Because of the dehydration and reaction with phosphoric acids, several low molecular acids formed large acidic oxygen-containing groups retained on the surface of the CLB.

The SEM graphs (Figure 2c,d) illustrated that CLB was mainly composed of carbon particles. It showed a relatively smooth surface with an irregular shape. These carbon particles vary in diameter from several to dozens of micrometers. Figure 2e,g depicted the morphology of CLB after 0.3 mM Cu(II) and 0.3 mM Pb(II) sorption, respectively. A mass of nano-sized particles occurred after Pb(II) sorption on CLB because Pb(II) precipitated onto the surface of carbon particles in aqueous solution. Corresponding EDX data of Figure 2h further proved that the lead element was detected on the surface of CLB after adsorption. In contrast, no precipitation particles were found on the surface of adsorbents after Cu(II) adsorption while EDX data (Figure 2f) showed that the copper element existed on it. This finding indicated that precipitation was not the main Cu(II) adsorption mechanism. Besides, EDX data also suggested that small amounts of phosphorus elements were detected after Cu(II)/Pb(II) adsorption, which probably derived from pristine CLB material.

To investigate the crystal structures of those materials before and after Cu(II)/Pb(II) adsorption, XRD experiments were performed, and the results were illustrated in Figure 3. The small amount of magnesium phosphate and silicon dioxide in the pattern of original CLB occurred and still existed after the adsorption (Figure 3b–d), certifying that these two substances are not directly involved in adsorption. Besides, no apparent shift of carbon peaks was found after adsorption, which indicated that the adsorption process had no significant impact on the carbon structure of the CLB. However, the peaks indexed to (Pb_5_(PO_4_)_3_OH) crystals were detected, which proved that Pb(II) was precipitated on the surface of CLB. Hence, the nano-sized particles mentioned above were probably Pb_5_(PO_4_)_3_OH precipitations, suggesting that sedimentation reactions were a significant mechanism during the Pb(II) adsorption process (Figure 3d). In contrast, no new peaks were found in CLB after adsorbing Cu(II) (Figure 3c). The result confirmed that precipitation was not the main mechanism for Cu(II) adsorption.

To further characterize the functional groups on the surface of CLB and post-sorption CLB, FTIR spectra were presented in Figure 4. The broad peak around 3428 cm^−1^ was assigned to the O–H group (usually from carboxyl) [33]. There was a weak band at 2852 cm^−1^ and a side sharper peak at 2921 cm^−1^, which were indicative of the deforming and stretching vibration of C–H bond, respectively [16]. The peaks at 1621 and 1701 cm^−1^ confirmed the stretching modes of carbon-carbon double bond (C=C) and carbonyl group (–COOH), respectively [34,35]. However, after Cu(II) and Pb(II) adsorption, the band at 3428 and 1701 cm^−1^ weaken, suggesting that O–H and –COOH functional groups participated in the adsorption. Hence, the amounts of surface functional groups could improve the adsorption property of CLB. The slight weakening of the band at 1620 cm^−1^ suggested the electrostatic attraction between CLB and metal ions [19]. The reaction equations were possible as follow:

2M–COOH + R^2+^→(M–COO)_2_R + 2H^+^ (3)

M–OH + R^2+^→M–OR^+^+H_3_O^+^ (4)

where M and R stand for the biochar surface and heavy metal ions, respectively.

A similar phenomenon was found with the peak centered 1220 cm^−1^, probably corresponding to the P=O, P–O–C, or P=OOH bond also weakening after adsorption [29]. The possible reason was that a phosphate crystal compound occurred after Pb(II) sorption, which highly agreed with XRD data and could be expressed in the following reaction equation:

(5) $$5{\mathrm{Pb}}^{2+}+3H_{2}{\mathrm{PO}}_{4}^{-}+H_{2}O\to {\mathrm{Pb}}_{5}({\mathrm{PO}}_{4}{)}_{3}\mathrm{OH}+7H^{+}$$

Besides, it has been reported that Cu(II) was more likely to be adsorbed on the surface of phosphoric acid activated carbons via complexation [19]. Another weak band at 1068 cm^−1^ could be considered as a phosphate ester bond (P^+^–O^−^) or polyphosphate chain (P–O–P). The band at 803 cm^−1^ represented the symmetric vibration of Si–H [36]. Hence, these newly-formed functional groups played a key role in metals sorption.

**Figure 5:**
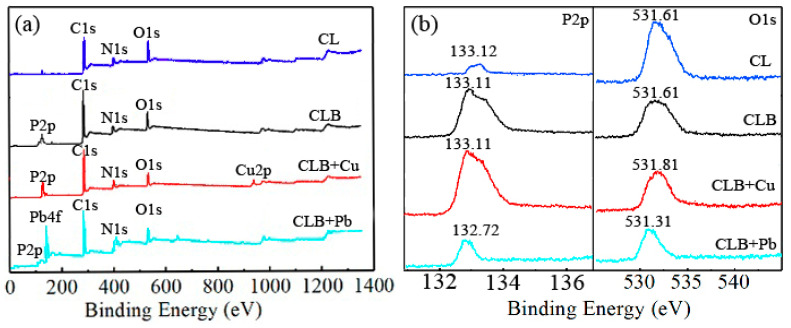
X-ray photoelectron spectra(XPS) of CL, CLB, and CLB before and after 0.3 mM Cu(II) and 0.3 mM Pb(II) adsorption, respectively. (**a**); XPS analysis of full spectrum; (**b**): P2p and O1s region.

To further investigate the preparation and adsorption mechanism, the surface elemental composition of CL, CLB, and CLB samples pre-and post-sorption were analyzed with XPS (Figure 5). The content (0.21%) of P in CL was less than that (1.77%) in CLB. Combined with the analysis of FTIR, the P-containing functional groups were successfully introduced into CLB after H_3_PO_4_ impregnation. For CLB, the content of Pb(II) was more than that of Cu(II) after sorption (Figure 5a), which was in line with the results of the sorption isotherms experiments discussed below. The comparisons indicated that CLB had a strong sorption capacity for Pb(II) relative to Cu(II). Furthermore, the peaks of O1s of CLB shifted after adsorption (Figure 5b). The phenomena confirmed that the O-containing functional groups participated in the sorption process, which was also following the result of FTIR. Besides, the P content of CLB decreased after Pb(II) sorption and the corresponding peak shifted (Figure 5b). The results suggested that the P-containing functional groups were related to Pb(II) sorption. By contrast, for Cu(II) sorption, the P-containing functional groups were not the main sorption sites. The results could be proved by the Pb_5_(PO_4_)_3_OH found by XRD mentioned above.

**Figure 6:**
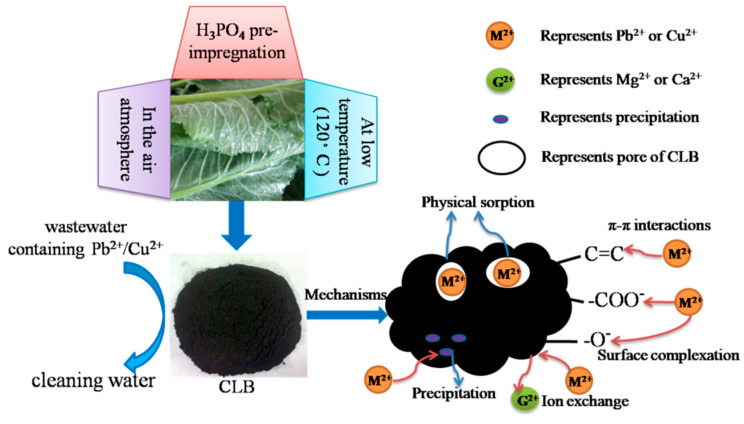
Summarized preparation process and mechanism of Cu(II) and Pb(II) sorption onto CLB.

The preparation process of CLB and its mechanisms of Cu(II) and Pb(II) sorption could be summarized in Figure 6, The main mechanisms included complexation (–OH and –COOH), chemical precipitation (Pb_5_(PO_4_)_3_OH), π–π interactions (C=C), physical sorption (pore-filling) and ion exchange.

### 3.3. Adsorption Kinetics and Isotherms

**Figure 7:**
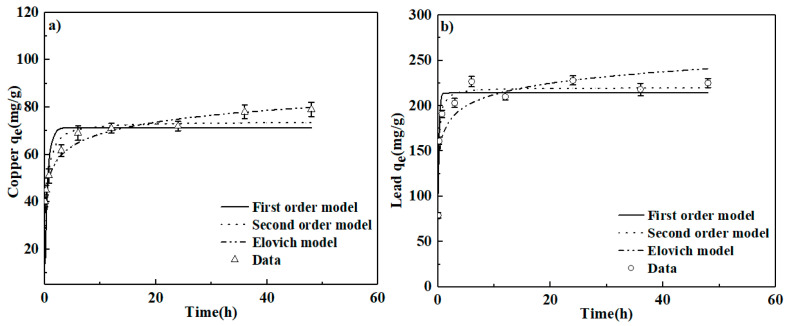

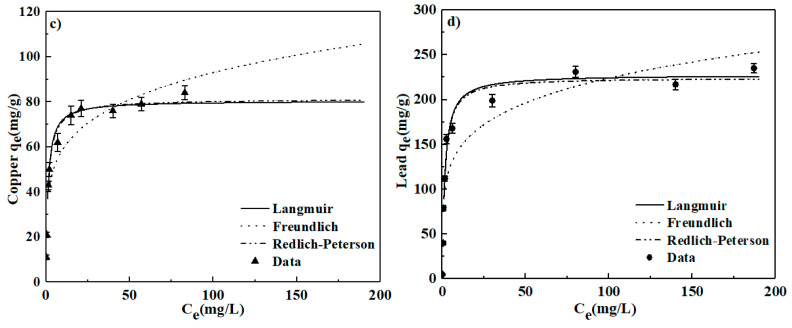
Kinetic (**a**,**b**) and isotherm (**c**,**d**) of Cu(II) and Pb(II) sorption on CLB at 25 ± 1 °C without pH modification. About kinetic experiments, samples were withdrawn at different time (0.05, 0.25, 0.75, 3, 6, 12, 24, 36, 48 h). (**a**): 0.25 g CLB was added into 100 mL solution containing 200 mg/L Cu(II); (**b**): 0.25 g CLB was added into 100 mL solution containing 600 mg/L Pb(II); As for isotherm experiments, samples were withdrawn after shaking 24 h. (**c**): 0.25 g CLB was added into 100 mL solution containing 30, 60, 90, 120, 150, 180, 210, 240, 270, 300 mg/L Cu(II), respectively. (**d**): 0.25 g CLB was added into 100 mL solution containing 50, 130, 210, 290, 370, 450, 530, 610, 690, 770 mg/L Pb(II), respectively.

To detect the sorption capacity of CLB, Cu(II) and Pb(II) adsorption kinetics were investigated (Figure 7a,b). The results showed that more than 50% sorption taken place rapidly during the initial contact time (0–4 h), and the equilibrium was reached at about 24 h. This kinetics sorption matched well with most trends of other biochars which showed that two phases exist during the whole sorption process, namely the early initial sorption phase and slow phase until equilibrium. Because there are sufficient sorption sites for Cu(II) and Pb(II) adsorption in the beginning. With the sorption process goes on, the sites gradually become saturated. The intra particle diffusion absorbed Cu (II) and Pb(II) into the interior surfaces of CLB and its micropores [18,37,38]. Pseudo-first-order, pseudo-second-order, and Elovich models were used to describe the sorption behavior of Cu(II) and Pb(II) onto CLB [13]:

(6) $$q_{t}=q_{e}(1-e^{{-k}_{1}t})$$

(7) $$q_{t}=\frac{k_{2}q_{e}^{2}t}{1+k_{2}q_{e}t}$$

(8) $$q_{t}=\frac{1}{\beta }\ln(\beta \alpha t+1)$$

where *q*_t_, *K*_1_, *q*_e_, *K*_2_, *α* and *β* are the amounts of sorbent adsorbed at any time t(mg/g), first-ordersorption rate constant (1/h), the amounts of sorbent adsorbed at equilibrium(mg/g), second-order sorption rate constant (g/(mg·min)), the initial Elovich sorption and desorption rate constant (g/mg), respectively.

The best-fitting model explaining the sorption of Pb(II) onto CLB was pseudo-second-order with the highest correlation coefficient (R^2^ = 0.978) (Table 2). Similar findings were also reported in previous literature [6,18]. However, the Elovich model can better describe the Cu(II) sorption (R^2^ = 0.945), compared with the other two models. This result further confirmed that chemisorptions participated in the process of Cu(II) sorption onto the heterogeneous surfaces via the surface complexation or ion exchange.

Langmuir, Freundlich and Redlich-Peterson models were used to further describe Cu(II) and Pb(II) sorption isotherms [13]:

(9) $$q_{e}=\frac{K_{L}C_{e}q_{m\mathrm{ax}}}{1+K_{L}C_{e}}$$

(10) $$q_{e}=K_{F}C_{e}^{1/n}$$

(11) $$q_{e}=\frac{KC_{e}^{r}q_{m\mathrm{ax}}}{1+KC_{e}^{r}}$$

where *C*_e_, *q*_max_, *K*_L_, *K*_F_, *n*, and r are the equilibrium concentration of metal ions (mg/L), the maximum adsorption capacity(mg/g), Langmuir equilibrium constant (L/g), Freundlich equilibrium constant (mg^(1–1/n)^·L^1/n^·g^−1^), and Freundlich and Redlich-Petersonlinearity constant, respectively.

The simulating results and related parameters (Figure 7c,d and Table 2) suggested that Redlich-Peterson model was most suitable for fitting Cu(II) and Pb(II) sorption (R^2^ ≥ 0.98), when compared to Langmuir (R^2^ < 0.98) and Freundlich (R^2^ < 0.90) models. These results indicated that Cu(II) and Pb(II) sorption was probably multilayer on a different surface, which might be caused by the fact that Redlich-Peterson isotherm is a combined model that describes the fitting of Langmuir and Freundlich models precisely. Each of these two models has only two parameters compared with the Redlich-Peterson model that has three parameters, leading to the highest R^2^ (better fitting). These simulating results were consistent with other previous studies [39]. Besides, the value of parameter “r” in the Redlich-Peterson model is commonly between 0 and 1. The closer the “r” value is to 0, the better the Freundlich model can describe the sorption process. Conversely, the near the “r” value is to 1, the better the Langmuir model can explain that. Hence, in this study, the obtained “r” values for Cu(II) and Pb(II) were both close to 1, accounting for that Langmuir model fitted better with experimental data than the Freundlich model. Based on the parameters of Redlich-Peterson model, the maximum sorption capacities for Cu(II) and Pb(II) were 81.43 and 224.60 mg/g, respectively, which kept higher performance of Cu(II) and Pb(II) sorption than many other types of biochars reported in the latest literature (Table 3). H_3_PO_4_ impregnation could improve biochar adsorption capacity by increasing surfaces P-containing and O-containing functional groups of CLB and its surface area [17,18,19]. Commonly, biochars prepared through pyrolysis demonstrated weak alkaline, but CLB in this study exhibited acidic. The reason was probably that more low molecular acids formed due to the acceleration of polysaccharides dehydration and degradation with H_3_PO_4_ impregnation. A large number of acidic O-containing groups was formed and retained on the surface of CLB [21]. Besides, moderate protons might catalyze the chemical reactions, which also enhanced the formation of acidic O-containing functional groups (e.g., –OH and –COOH) [17]. Moreover, the cross-linking reaction enhanced by H_3_PO_4_ could help forming P-containing structures on the surface of CLB. In addition to these reasons, the different pristine material was also one of the critical factors determining the adsorption capacity of biochars, because the biochars derived from different biomass sources had various organic functional groups and surface area. H_3_PO_4_ could easily react with these functional groups and release the CO_2_ and H_2_O (g) [18,19]. Then lots of pores were created and SSA of biochars enlarged, which explained that SSA of CLB was much higher than that of CL and biochars produced from different biomass sources varied in terms of SSA [19]. Therefore, CLB had relatively excellent Cu(II) and Pb(II) sorption behavior, although their sorption capacities of CLB were less than that of several biochars reported in previous research. The comparison results also suggested that this prepared method at low temperature in the air atmosphere was beneficial and low-cost. Through this method, transforming the waste vegetable leaves into efficient sorbents was feasible for heavy metal contaminated water remediation.

### 3.4. pH Influence

**Figure 8:**
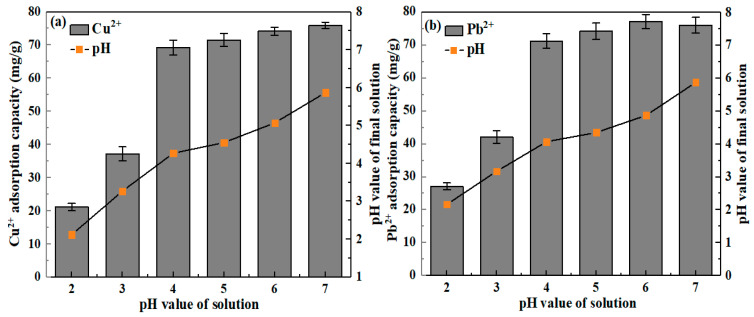
Effect of pH value on 200 mg/L Cu(II) (**a**) and 200 mg/L Pb(II) (**b**) sorption onto CLB, respectively. (adsorbent dose: 0.5 g; solution volume: 200 mL; temperature: 25 ± 1 °C; contact time: 24 h).

With the increase of initial pH value, the sorption capacities of Cu(II) onto CLB improved (Figure 8a). A little sorption capacity (21.12 mg/g and 37.17 mg/g) of Cu(II) was found at pH 2 and 3, suggesting that low removal rate behavior of CLB at lesser pH. This result was in consistent with those in previous pieces of literature [45,46]. The sorption capacity jumped into 69.27 mg/g at pH 4 and increased to reach 74.21 and 75.97 mg/g at pH 6 and 7, respectively. The reason for these results was that a huge amount of protonation of CLB surface could greatly affect the sorption with solution pH decrease, since abundance H_3_O^+^ ions compete for the binding sites and then prevent the formation of compounds of Cu(II). By contrast in high pH solution, the more negative charge was formed by the deprotonation of CLB, favoring metal ions sorption by electrostatic interaction. Cu(II) sorption onto CLB was not markedly influenced via protonation interaction except that solution pH **≤** 3. The result explained that, besides electrostatic interaction, multiple sorption reactions (Equations (2)–(4)) existed [47]. Moreover, the similar sorption trend that CLB had 42.17 and 71.27 mg/g of Pb(II) sorption capacity were found at pH 3 and 4, respectively (Figure 8b). Unlike trend of pH influence for Cu(II) sorption, Pb(II) sorption capacity reduced slightly from 77.21 mg/g (pH = 6) to 76.07 mg/g (pH = 7). In a word, CLB demonstrated efficient and stable Cu(II) and Pb(II) sorption capacity and its sorption mechanism should be dominated by a chemical reaction when the initial solution pH was more than 4.

### 3.5. Competitive Sorption

**Figure 9:**
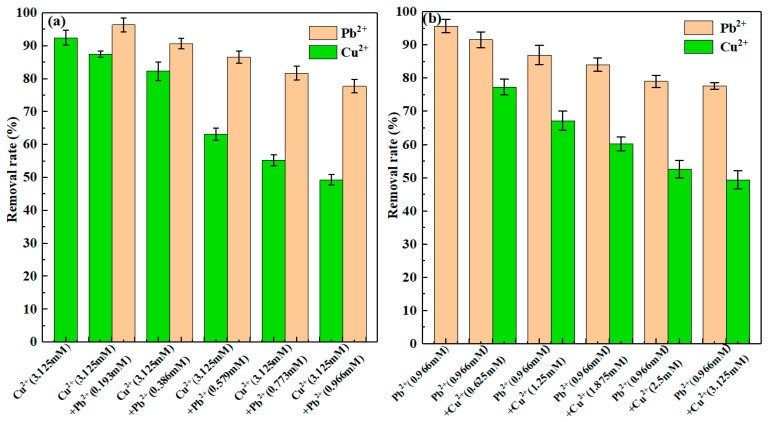
Removal rate of competitive adsorption of Cu(II)and Pb(II) onto CLB with 3.125 mM Cu(II) (**a**) and 0.966 mM Pb(II) (**b**) as background solution, respectively, at pH = 6.

The competitive sorption results were presented in Figure 9. With 200 mg/L (3.125 mM) Cu(II) as the background solution (Figure 9a), a slight competitive relationship existed between Pb(II) and Cu(II) when Pb(II) concentration was less than or equal to 80 mg/L (0.386 mM). The reason was possible that CLB had a strong sorption capacity. Conversely, when the Pb(II) concentration was over 80 mg/L (0.386 mM), the increase of Pb(II) significantly reduces the Cu(II) adsorption capacity. The results indicated that Pb(II) and Cu(II) may have similar adsorption mechanisms and have a relatively fierce competitive relationship. Interestingly, compared with the removal rate in a single solution, the removal rates in dual-metals solution decreased significantly in the mixed solutions. Possibly because the adsorption site of CLB was saturated. Besides, with 200 mg/L (0.966 mM) Pb(II) as background solutions (Figure 9b), the competitive adsorption data also verified the above competitive relationship. It could be observed that the increase of Cu(II) concentration reduced the Pb(II) adsorption capacity. However, compared with Figure 9a, Pb(II) had a more significant impact on Cu(II) adsorption, confirming that Pb(II) was easier to adsorb and had a higher adsorption priority. This research result was in accord with previous literature about biochars prepared from banana peels [13], poly(acrylic acid) grafted chitosan, and rice straw biochar composite [48]. The result was also supported by EDS spectra (Figure 2f,h), which demonstrated a higher peak of Pb(II) sorption than that of Cu(II) sorption.

Many factors (e.g., metal electronegativity, hydrate ion radius, and charge carried by mental ions) have a great influence on this competitive sorption [49]. As Pb(II) electronegativity (2.33) is higher than Cu(II) electronegativity (1.90), Pb(II) might have stronger sorption potential than Cu(II). In addition, commonly the higher ionic radius (ionic radius of Pb(II) = 122 p.m.; ionic radius of Cu(II) = 72 p.m.) means the lower hydrated radius (hydrated radius of Pb(II) = 261 p.m.; hydrated radius of Cu(II) = 295 p.m.) and the lower hydrated radius might intensify the Pb(II) competitive sorption [13]. Another reason is that Pb(II) has a higher precipitation potential than Cu(II) because solubility product (K_SP-Pb_ = 8 × 10^−43^) of Pb(II) precipitate with ${\mathrm{PO}}_{4}^{3-}$ is smaller than that (K_SP-Cu_ = 1.3 × 10^−37^) of Cu(II)precipitate [50]. With respect to the complexation mechanisms, Pb(II) has the very higher ionic potential than Cu(II) ($\sqrt{\phi }$Pb(0.22)>$\sqrt{\phi }$Cu(0.10)) [51]. Therefore, Pb(II) had stronger complexation with –O^−^, which showed less inhibition by Cu(II).

### 3.6. Reusability of CLB

**Figure 10:**
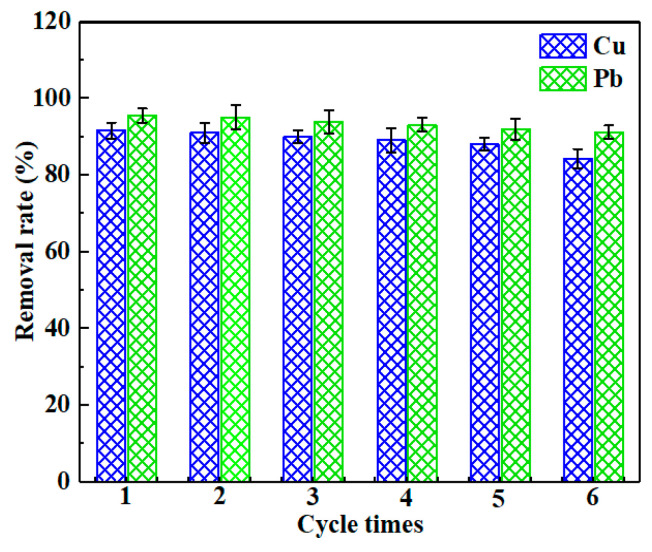
Reusability of CLB for Cu(II) and Pb(II) adsorption (adsorbent dose: 0.25 g; solution volume: 100 mL; temperature: 25 ± 1 °C; contact time: 24 h; initial concentration: 200 mg/L Cu(II)/ 600 mg/L Pb(II)).

The reusability of CLB for the removal of Cu(II) and Pb(II) was assessed via six successive sorption and desorption cycles. According to the previous discussion (Section 3.4.), the adsorption sites of CLB became sensitive to hydrogen ion when solution pH ≤ 4. So 0.1 mol/L HNO_3_ solution was selected as the desorption solution to desorb the heavy metals adsorbed onto the surface of CLB. Figure 10 presented the result of sorption and desorption cycles. No obvious decline in the sorption capacities of Cu(II) and Pb(II) on the CLB was found in the six successive cycles. The sorption capacities of CLB for Cu(II) and Pb(II) removal were still maintained above 84% and 91%, respectively, after six successive cycles, explaining that CLB had the high stability to be industrially used in the future.

## 4. Conclusions

An economic and environmentally friendly method that effectively introduced phosphorous and oxygenic groups onto biochar surface at extremely low temperature (120 °C) in the air atmosphere was successfully adopted to prepare CLB. The synthesized CLB had an excellent Cu(II) and Pb(II) sorption performance because there was a mass of–COOH and –OH groups on its surface. Besides, the newly-formed phosphorus-containing groups could enhance Cu(II) and Pb(II) sorption. During Pb(II) sorption process, Pb_5_(PO_4_)_3_OH crystals occurred and precipitated on the surface of CLB. Additionally, the sorption kinetic experiments suggested that the sorption process of Cu(II) and Pb(II) sorption were well described by the Elovich model and pseudo-second-order model, respectively. The isotherm experiments demonstrated that the sorption behaviors of these two metals were satisfactorily fitted with Redlich-Peterson model. The maximum adsorption capacities for Cu(II) and Pb(II) calculated by Redlich-Peterson model were 81.43 and 224.60 mg/g, respectively. The solution pH ranged from 4 to 7 had little impact on Cu(II) and Pb(II) sorption and competitive sorption confirmed that Pb(II) had a higher adsorption priority than Cu(II). This work indicated that CLB was an eco-friendly and low-cost sorbent. Considering its stability and efficiency, CLB had a great potential application for the removal of heavy metals from aqueous solutions.

## Figures and Tables

**Table 1:** Elemental composition, pH, SSA, and pH_zpc_ values of pristine cauliflower leaves(PCL), cauliflower leaves(CL), and cauliflower leaves biochar (CLB).

Header	PCL	CL	CLB
C (%)	9.65	59.71	63.39
O (%)	78.17	23.57	14.43
H (%)	10.74	7.02	5.42
S (%)	0.29	0.27	0.31
N (%)	0.32	1.79	1.92
P (%)	0.025	0.21	1.77
O/C	8.101	0.395	0.228
H/C	1.113	0.118	0.086
(O + H)/C	9.213	0.425	0.258
pH	—	6.47	4.56
SSA(m^2^/g)	—	<5	55.42
pH_zpc_	—	5.84	3.27
Ash (%)	—	7.24	9.97

**Table 2:** Fitting parameters of kinetic models and isotherm models for Cu(II) and Pb(II) sorption onto CLB.

Element	Model	CLB Model Parameters		
		Parameter 1	Parameter 2	R^2^
Kinetic models				
	First-order	*q*_e_ = 71.26	*K*_f_ = 2.177	0.690
Cu	Second-order	*q*_e_ = 73.91	*K*_2_ = 0.047	0.847
	Elovich	*α* = 10007.19	*β* = 0.139	0.945
	First-order	*q*_e_ = 214.12	*K*_f_ = 6.579	0.914
Pb	Second-order	*q*_e_ = 220.11	*K*_2_ = 0.0486	0.978
	Elovich	*α* = 225981.54	*β* = 0.0553	0.806
Isotherm models				
	Langmuir	*q*_max_ = 80.41	*K*_L_ = 0.863	0.972
Cu	Freundlich	*n* = 5.034	*K*_f_ = 37.24	0.866
	Redlich-Peterson	*q*_max_ = 81.43	*K* =0.840 r = 0.928	0.980
	Langmuir	*q*_max_ = 227.80	*K*_L_ = 0.658	0.971
Pb	Freundlich	*n* = 5.247	*K*_f_ = 93.42	0.847
	Redlich-Peterson	*q*_max_ = 224.60	*K* = 0.660 r = 1.02	0.981

**Table 3:** Comparisonof Cu(II) and Pb(II) adsorption capacity of CLB with other biochars.

Biosorbents	Preparation Method and Condition	Cu(II) Sorption Capacity (mg/g)	Pb(II) Sorption Capacity (mg/g)	Reference
H_3_PO_4_-modified banana peel biochar	Hydrothermal carbonization, anaerobism, 230 °C for 2 h	— ^a^	241	[17]
H_3_PO_4_-modified chicken feather biochar	Pyrolysis, anaerobism, 450 °C, 1 h	— ^a^	77.46	[18]
H_3_PO_4_-modified pine sawdust biochar	Pyrolysis, anaerobism, 350 °C, 2 h	about 30	— ^a^	[19]
ferromanganese binary oxide–corn straw biochar	Pyrolysis, anaerobism, 600 °C, 2 h	64.9	— ^a^	[40]
HCl modified Phoenix Dactylifera biochar	Pyrolysis, anaerobism, 550 °C, 3 h	45.12	188.58	[41]
Gingko leaf biochar	Pyrolysis anaerobism, 800 °C, 1.5 h	59.9	138.9	[42]
Enteromorpha biochar	Hydrothermal carbonization anaerobism, 250 °C, 40 min	98	254	[43]
Medulla tetrapanacis biochar	Pyrolysis anaerobism, 400 °C, 1 h	168.35	458.72	[44]
carboxyl-modified Palm fiber biochar	Pyrolysis anaerobism, 400 °C, 2 h	132.1	218.2	[33]
H_3_PO_4_ impregnation cauliflower leaves biochar	Hydrothermal carbonization, In air atmosphere, 120 °C, 2 h	81.43	224.6	This work

^a^ not reported.
